# Exploring the risk of second primary malignancies in laryngeal cancer survivors: insights from the SEER database

**DOI:** 10.1007/s00405-024-08731-9

**Published:** 2024-05-22

**Authors:** Abdelrahman Yousry Afify, Mohamed Hady Ashry

**Affiliations:** 1 School of Medicine, New Giza University (NGU), Giza, Egypt; 2 Medical Research Platform, Cairo, Egypt

**Keywords:** Laryngeal cancer, Larynx, Cancer, Second primary malignancy, SEER, Surveillance

## Abstract

**Purpose:**

We intended to investigate the risk for second primary malignancy (SPM) development in Laryngeal Cancer (LC) survivors. We conducted a population-based analysis of SPM risk using the National Cancer Institute’s Surveillance, Epidemiology, and End Results (SEER) database.

**Methods:**

Data of selected LC survivors from the SEER database between 2000 and 2020 were examined. Standardized Incidence ratios (SIRs) for SPM development were calculated, followed by detailed stratification according to anatomical site and different latency periods.

**Results:**

A total of 8413 SPMs were observed in our extracted cohort. The collective standardized incidence of SPMs was 2.12 (95% CI 2.07–2.17) compared to the US population, with an absolute excess risk (AER) of 201.73 per 10,000 individuals. The highest SPM risks were observed in patients with young age at diagnosis, females, and American Indians/Alaska natives. Increased SPM risks were reported in patients receiving all modalities of treatment including surgery, chemotherapy, and radiotherapy. Most SPMs were detected in solid organs such as the lungs and bronchus, oral cavity and pharynx, and prostate. The highest increased risks of developing SPMs were observed in Trachea, larynx, oral cavity and pharynx, lung and bronchus, and esophagus.

**Conclusions:**

The risk of SPMs in LC survivors was significantly increased compared to the general US population. Accordingly, a more impactful cancer surveillance strategy for LC patients should be implemented.

## Background

Laryngeal cancer (LC) is one the most prevalent head and neck malignancies, accounting for more than 184,000 new cases yearly worldwide [1]. Additionally, approximately 100,000 LC deaths are recorded annually according to the World Health Organization [1]. In the United States alone, more than 12,000 new cases of LC have been recorded and approximately 4000 of these patients died [2]. Tobacco use is considered to be the main risk factor for LC development, and the relative risk remains elevated during the first 15 years of quitting smoking [3]. The survival time of patients with LC has been significantly prolonged in recent decades owing to early diagnosis and advancements in treatment. This results in a prolonged exposure period for second primary malignancy (SPM). The current 5-year disease-free survival rate for patients with non-metastatic LC is more than 61% [4]. In our previous work, we observed an increased risk of death attributed to secondary cancers in LC patients which further solidifies the need for investigating the incidence of SPMs in this vulnerable patient population [5].

To date, studies on the risk of SPM development in LC are scarce and outdated. Our study aims to explore the risk of developing SPMs in LC survivors in recent years. We utilized the National Cancer Institute’s Surveillance, Epidemiology, and End Results (SEER) database to calculate the standardized incidence ratios of SPMs in patients with LC compared to the general US population.

## Methods

### Study design

We conducted a retrospective, observational cohort study in accordance with the Strengthening the Reporting of Observational Studies in Epidemiology (STROBE) guidelines.

### Data source and study population

The Surveillance, Epidemiology, and End Results (SEER) program was used to conduct this study. Using the SEER*Stat software, we extracted the data of cancer patients from 2000 to 2020 from the SEER 17 registries covering approximately (26.5%) of the US population [6, 7]. Institutional review board approval was not required for this study because the SEER data is anonymized and considered non-human research.

### Study population

We included all patients diagnosed with laryngeal cancer with confirmed malignant behavior between 2000 and 2020 in the United States and followed them until death or the end of 2020. LC cases were identified using “site and morphology” recode based on the ICD-0-3/WHO 2008 definitions. Patients identified through death certificates and autopsies were excluded from the analysis.

### Study outcomes

Our main study outcome was the standardized incidence of developing a second cancer after a diagnosis of LC compared to the general US population. We used an exclusion latency period of 6 months from the time of LC diagnosis, disregarding possible incidental synchronous cancers. For our cohort, we analyzed the incidence of secondary malignancy with respect to different demographic and tumor characteristics, including sex, race, age at diagnosis, disease stage, tumor grade, histological subtype, and treatment modality. The risk of developing secondary primary cancers was further explored with detailed stratification according to the anatomical site of the SPMs. We also considered different latency periods from the time of diagnosis to SPM detection. We stratified SPM risk into four different periods: 6–11 months, 1–5 years, 5–10 years, and > 10 years after LC diagnosis.

### Statistical analysis

We calculated the standardized incidence ratio (SIR) for each reported SPM in our cohort. SIRs were defined as the observed-to-expected ratio (O/E), where “observed” represents the number of SPMs experienced by LC patients, and “expected” represents the number of people who are expected to develop malignancy in a demographically similar general population. We also calculated the absolute excess ratios (AERs) per 10,000 individuals. We estimated SIRs with 95% confidence intervals (CIs) and AERs using the SEER*Stat software (version 8.4.3). Multiple outcome analysis was used throughout the study as recommended to capture the incidence of SPMs over the entire course of the patients’ follow-up period. A *p*-value of less than 0.05 was considered statistically significant.

## Results

### Baseline characteristics

Data of 40,023 patients diagnosed with LC between 2000 and 2020 were collected and analyzed. Most of the patients were between the ages of 50 and 74 at diagnosis (73.5%), white (81.5%), males (80.9%), and had localized disease (51.7%). Squamous cell origin was the dominant histological subtype, diagnosed in 96.6% of patients. 31.7% and 78.5% of the entire cohort received chemotherapy and radiotherapy, respectively. Over the follow-up period, a total of 8413 SPMs were reported. The mean age at diagnosis of the index LC was 64 years, whereas the mean age at SPM was approximately 70 years. The standardized incidence of SPMs in the overall cohort compared to the US population was 2.12 (95% CI 2.07–2.17), with an AER of 201.73 per 10,000 individuals (Table 1).

**Table 1:** Baseline characteristics of patients with laryngeal cancer and their corresponding standardized incidence ratios (SIRs) and absolute excess risks (AER)s

Characteristic	Total no. of patients (%)	No. of SPMs (%)	SIRs (95% CI)	Absolute excess risk	Mean age at index cancer, y	Mean age at SPM, y
Overall	40,023 (100)	8413 (100)	2.12 (2.07–2.17)^a^	201.73	64.06	69.9
Age at diagnosis, y						
0–49	3880 (9.69)	599 (7.12)	3.64 (3.35–3.94)^a^	146.9	44.52	53.46
50–74	29,442 (73.56)	6642 (78.95)	2.17 (2.12–2.23)^a^	218.36	62.8	68.91
> 74	6701 (16.74)	1172 (13.93)	1.57 (1.48–1.66)^a^	160.04	80.91	83.9
Sex						
Male	32,373 (80.89)	6873 (81.69)	1.99 (1.94–2.03)^a^	190.38	64.29	70.12
Female	7650 (19.11)	1540 (18.31)	3.02 (2.87–3.18)^a^	251.36	63.07	68.88
Race						
White	32,638 (81.55)	6973 (82.88)	2.1 (2.05–2.15)^a^	200.26	64.38	70.23
Black	5792 (14.47)	1165 (13.85)	2.19 (2.07–2.32)^a^	220.11	61.73	67.56
Asian or Pacific Islander	1413 (3.53)	242 (2.88)	2.27 (1.99–2.58)^a^	162.73	66.53	72.02
American Indian/Alaska Native	180 (0.45)	33 (0.39)	3.81 (2.62–5.35)^a^	272.18	62.07	66.55
Disease stage						
Localized	20,720 (51.77)	5367 (63.79)	1.9 (1.85–1.95)^a^	171.28	65.24	71.53
Regional	7714 (19.27)	1627 (19.34)	3.06 (2.91–3.21)^a^	313.78	61.71	66.3
Distant	5817 (14.53)	954 (11.34)	2.58 (2.42–2.74)^a^	248.78	61.79	67.32
Unknown	1443 (3.61)	285 (3.39)	1.74 (1.54–1.95)^a^	137.97	65.32	70.12
Blank	4329 (10.82)	180 (2.14)	2.35 (2.02–2.72)^a^	215.17	65.25	66.96
Tumor grade						
Well-differentiated: Grade 1	5106 (12.76)	1175 (13.97)	1.72 (1.63–1.82)^a^	134.88	64.26	70.98
Moderately differentiated: Grade 2	16,482 (41.18)	3928 (46.69)	2.2 (2.13–2.27)^a^	214.17	63.65	69.65
Poorly differentiated: Grade 3	5957 (14.88)	1294 (15.38)	2.54 (2.4–2.68)^a^	265.33	63.58	68.72
Undifferentiated, anaplastic: Grade 4	252 (0.63)	53 (0.63)	2.5 (1.87–3.26)^a^	272.39	65.03	72.5
Unknown	12,226 (30.55)	1963 (23.33)	2.03 (1.94–2.12)^a^	187.41	64.74	70.44
Histological subtype						
Unspecified	205 (0.51)	28 (0.33)	2.16 (1.44–3.13)^a^	192.36	66.47	70
Epithelial NOS	621 (1.55)	109 (1.30)	1.99 (1.63–2.4)^a^	192.48	65.24	72.93
Squamous	38,683 (96.65)	8200 (97.47)	2.13 (2.08–2.17)^a^	203.47	64.07	69.88
Transitional cell	11 (0.03)	1 (0.01)	2.17 (0.05–12.09)	229.5	67.09	57.33
Adenocarcinoma	195 (0.49)	21 (0.25)	1.56 (0.97–2.38)	68.39	59.46	67.29
Mucoepidermoid neoplasm	17 (0.04)	1 (0.01)	0.9 (0.02–5)	-11.27	57.82	71.75
Complex epithelial neoplasm	45 (0.11)	12 (0.14)	3.94 (2.04–6.89)^a^	532.84	64.53	67.65
Nevi/melanoma	3 (0.01)	1 (0.01)	13.38 (0.34–74.55)	1261.93	58.97	62
Soft tissue sarcomas	29 (0.07)	8 (0.10)	2.31 (1–4.55)	279.56	70.61	64.41
Myomatous neoplasm	15 (0.04)	4 (0.05)	3.66 (1–9.38)	280.41	51.1	60.93
Complex, mixed, and stromal neoplasms	21 (0.05)	5 (0.06)	1.85 (0.6–4.32)	170.12	65.88	69.45
Osseous and chondromatous neoplasms	134 (0.33)	23 (0.27)	1.39 (0.88–2.08)	60.71	60.84	68.22
Chemotherapy						
No/unknown	27,327 (68.28)	5961 (70.85)	1.89 (1.84–1.94)^a^	167.92	65.35	71.22
Yes	12,696 (31.72)	2452 (29.15)	3.01 (2.89–3.13)^a^	307.97	61.28	66.67
Radiotherapy						
No/unknown	8180 (20.44)	1479 (17.58)	1.78 (1.69–1.88)^a^	140.83	64.47	70.87
Refused	395 (0.99)	44 (0.52)	2.14 (1.56–2.88)^a^	202.03	66.59	70.87
Yes	31,448 (78.57)	6890 (81.90)	2.21 (2.16–2.26)^a^	217.99	63.92	69.68

^a^p value < 0.05

### SPMs risk by baseline characteristics

Regarding baseline demographics, all patients diagnosed with LC had a statistically significant increased risk of developing SPMs during their survival period, regardless of their age at diagnosis, sex, and race. However, the highest risks were observed in younger patients (SIR = 3.64, 95% CI 3.35–3.94) with a decreasing trend in older patients at diagnosis, females (SIR = 3.02, 95% CI 2.87–3.18), and American Indians/Alaska natives (SIR = 3.81, 95% CI 2.62–5.35). Moreover, patients diagnosed with localized, regional, and distant disease had significant SIRs of 1.9 (95% CI 1.85–1.95), 3.06 (95% CI 2.91–3.21), and 2.58 (95% CI 2.42–2.74), respectively. Regarding the histological subtype of LC, patients with squamous cell carcinoma, unspecified epithelial carcinomas, and those labelled as complex epithelial tumors had an increased risk of developing SPMs (SIR = 2.13, SIR = 1.99, and SIR = 3.94, respectively). However, patient groups with other histological subtypes failed to reach statistical significance. Interestingly, all the treatment-stratified groups exhibited increased risk of developing secondary tumors including those who received chemotherapy (SIR = 3.01, 95% CI 2.89–3.13) and radiotherapy (SIR = 2.21, 95% CI 2.16–2.26) and patients who did not receive chemotherapy (SIR = 1.89, 95% CI 1.84–1.94) and radiotherapy (no/unknown, SIR = 1.78, 95% CI 1.69–1.88 and refused, SIR = 2.14, 95% CI 1.56–2.88) (Table 1).

### SPMs risk by anatomical site

Overall, SPMs in LC patients were mostly detected in solid organs (n = 7757; 92.2%). The distinct sites with the highest number of reported SPMs were the lung and bronchus (n = 3130; 37.2%), oral cavity and pharynx (n = 879; 10.4%), prostate (n = 868; 10.3%), colon and rectum (n = 500; 5.9%), and larynx (n = 419; 5.0%). The highest increased risks of developing SPMs were observed in Trachea (SIR = 72.01, 95% CI 49.87–100.62, AER = 1.52), larynx (SIR = 10.61, 95% CI 9.61–11.67, AER = 17.22), oral cavity and pharynx (SIR = 8.02, 95% CI 7.5–8.57, AER = 34.92), lung and bronchus (SIR = 5.5, 95% CI 5.31–5.69, AER = 116.22), and esophagus (SIR = 5.38, 95% CI 4.78–6.03, AER = 10.94). Interestingly, SPMs of the urinary system had increased incidence that was mainly derived by cancers of the urinary bladder (SIR = 1.52, 95% CI 1.37–1.67, AER = 6.32) and not renal tumors (SIR = 1.07, 95% CI 0.91–1.25, AER = 0.5). In contrast, a decreased risk of SPMs arising from the prostate, brain/CNS, and skin was observed in LC patients (SIR = 0.9, 95% CI 0.84–0.96, AER = − 4.4, SIR = 0.65, 95% CI 0.43–0.96, AER = − 0.62, and SIR = 0.77, 95% CI 0.66–0.89, AER = − 2.34, respectively) (Table 2).

**Table 2:** The standardized incidence ratios (SIRs) and absolute excess risks (AER)s of second primary malignancies (SPMs)in laryngeal cancer by anatomical site

SPM site	All		
	Observed	SIR (95% CI)	AER
All sites	8413	2.12 (2.07–2.17)^a^	201.73
All solid tumors	7757	2.26 (2.21–2.31)^a^	196.54
Oral cavity and pharynx	879	8.02 (7.5–8.57)^a^	34.92
Esophagus	296	5.38 (4.78–6.03)^a^	10.94
Stomach	102	1.43 (1.16–1.73)^a^	1.39
Small intestine	20	1 (0.61–1.54)	0
Colon and rectum	500	1.43 (1.31–1.56)^a^	6.83
Anus, anal canal and anorectum	30	2.73 (1.84–3.89)^a^	0.86
Liver, gallbladder, intrahepatic bile duct, and other biliary	175	1.51 (1.3–1.75)^a^	2.69
Pancreas	142	1.18 (1–1.39)	0.99
Nose, nasal cavity, and middle ear	18	3.07 (1.82–4.85)^a^	0.55
Larynx	419	10.61 (9.61–11.67)^a^	17.22
Lung and bronchus	3130	5.5 (5.31–5.69)^a^	116.22
Trachea	34	72.01 (49.87–100.62)^a^	1.52
Bones and joints	7	1.87 (0.75–3.85)	0.15
Soft tissue including heart	37	1.6 (1.13–2.21)^a^	0.63
Skin excluding basal and squamous	170	0.77 (0.66–0.89)^a^	− 2.34
Breast	147	0.96 (0.81–1.13)	− 0.29
Female genital system	60	1.06 (0.81–1.36)	0.15
Male genital system (combined)	879	0.9 (0.84–0.96)^a^	− 4.41
Prostate	868	0.9 (0.84–0.96)^a^	− 4.4
Urinary system (combined)	585	1.36 (1.25–1.48)^a^	7.05
Urinary bladder	409	1.52 (1.37–1.67)^a^	6.32
Kidney and renal pelvis	160	1.07 (0.91–1.25)	0.5
Eye and orbit	12	1.97 (1.02–3.43)^a^	0.27
Brain and other nervous system	26	0.65 (0.43–0.96)^a^	− 0.62
Endocrine system	79	1.89 (1.5–2.36)^a^	1.69
Thyroid	76	2 (1.58–2.51)^a^	1.73
All lymphatic and hematopoietic diseases (combined)	376	1.04 (0.94–1.15)	0.7
Lymphoma	194	1.12 (0.97–1.29)	0.96
Hodgkin–extranodal	0	0 (0–13.89)	− 0.01
Non-Hodgkin lymphoma	188	1.14 (0.99–1.32)	1.07
Myeloma	60	0.87 (0.66–1.12)	− 0.41
Lymphocytic leukemia	45	0.75 (0.55–1)	− 0.69
Non-lymphocytic leukemia	77	1.31 (1.04–1.64)^a^	0.84
Mesothelioma	15	1.16 (0.65–1.92)	0.09
Miscellaneous	257	1.6 (1.41–1.8)^a^	4.36

^a^p value < 0.05

### SPMs risk by latency

Patients with LC exhibited an elevated incidence of overall SPMs occurrence during the first year (6–11 months) following diagnosis (SIR = 2.11, 95% CI 1.95–2.28, AER = 181.89), 1–5 years following diagnosis (SIR = 2.12, 95% CI 2.05–2.19, AER = 193.05), 5–10 years following diagnosis (SIR = 2.23, 95% CI 2.15–2.32, AER = 229.69), and greater than 10 years following diagnosis (SIR = 1.93, 95% CI 1.83–2.04, AER = 185.36). The highest total number of developed SPMs was observed in the 1–5 years period (n = 3712), with a decreasing trend towards the later periods (5–10 years n = 2715; and > 10 years, n = 1337). However, the greatest number of SPMs per month was reported in the first year after LC diagnosis (6–11 months). Regarding the anatomical sites of SPMs, the distribution across the four examined time periods generally closely followed the overall cohort, although some sites failed to reach statistical significance in certain periods, owing to the low number of observed SPMs (Table 3).

**Table 3:** The standardized incidence ratios (SIRs) and absolute excess risks (AER)s of second primary malignancies (SPMs) in laryngeal cancer by latency periods

SPM site	6–11 months			1–5 years			5–10 years			> 10 years		
	Observed	SIR (95% CI)	AER	Observed	SIR (95% CI)	AER	Observed	SIR (95% CI)	AER	Observed	SIR (95% CI)	AER
All sites	649	2.11 (1.95–2.28)^a^	181.89	3712	2.12 (2.05–2.19)^a^	193.05	2715	2.23 (2.15–2.32)^a^	229.69	1337	1.93 (1.83–2.04)^a^	185.36
All solid tumors	602	2.24 (2.07–2.43)^a^	177.68	3429	2.25 (2.18–2.33)^a^	187.77	2508	2.4 (2.3–2.49)^a^	224.04	1218	2.07 (1.96–2.19)^a^	180.8
Oral cavity and pharynx	57	6.68 (5.06–8.66)^a^	25.83	332	6.89 (6.17–7.67)^a^	27.96	338	10.08 (9.03–11.22)^a^	46.69	152	7.87 (6.67–9.23)^a^	38.08
Esophagus	25	5.93 (3.83–8.75)^a^	11.08	118	4.89 (4.05–5.86)^a^	9.25	97	5.72 (4.64–6.98)^a^	12.28	56	5.74 (4.34–7.46)^a^	13.27
Stomach	9	1.65 (0.75–3.13)^a^	1.89	44	1.41 (1.02–1.89)^a^	1.26	31	1.41 (0.96–2)	1.37	18	1.41 (0.84–2.23)	1.5
Small intestine	1	0.68 (0.02–3.78)	− 0.25	8	0.94 (0.4–1.84)	− 0.05	7	1.12 (0.45–2.3)	0.11	4	1.06 (0.29–2.71)	0.06
Colon and rectum	59	2.07 (1.58–2.67)	16.26	213	1.34 (1.17–1.53)^a^	5.32	146	1.39 (1.17–1.63)^a^	6.23	82	1.45 (1.15–1.79)^a^	7.26
Anus, anal canal and anorectum	2	2.35 (0.28–8.5)	0.61	19	3.94 (2.37–6.16)^a^	1.4	7	2.08 (0.84–4.29)	0.56	2	1.01 (0.12–3.65)	0.01
Liver, gallbladder, intrahepatic bile duct, and other biliary	15	1.78 (1–2.93)	3.5	67	1.36 (1.06–1.73)^a^	1.76	71	1.96 (1.53–2.47)^a^	5.32	22	1 (0.63–1.52)	0.03
Pancreas	11	1.28 (0.64–2.29)	1.28	65	1.29 (0.99–1.64)	1.43	46	1.22 (0.89–1.63)	1.27	20	0.86 (0.52–1.32)	− 0.96
Nose, nasal cavity, and middle ear	0	0 (0–8.27)	− 0.24	8	3.13 (1.35–6.16)^a^	0.54	4	2.21 (0.6–5.66)	0.34	6	5.71 (2.1–12.43)^a^	1.42
Larynx	7	2.15 (0.86–4.43)	2	116	6.44 (5.32–7.73)^a^	9.65	196	16.47 (14.25–18.95)^a^	28.23	100	15.76 (12.82–19.17)^a^	26.88
Lung and bronchus	234	5.3 (4.64–6.03)^a^	101.19	1517	6.03 (5.73–6.34)^a^	124.66	952	5.44 (5.1–5.8)^a^	119.18	427	4.32 (3.92–4.75)^a^	94.2
Trachea	4	103.81 (28.28–265.78)^a^	2.11	19	89.52 (53.9–139.8)^a^	1.85	9	63.35 (28.97–120.26)^a^	1.36	2	25.21 (3.05–91.07)^a^	0.55
Bones and joints	0	0 (0–12.92)	− 0.15	1	0.61 (0.02–3.42)	− 0.06	4	3.47 (0.94–8.87)	0.44	2	2.94 (0.36–10.62)	0.38
Soft tissue including heart	1	0.59 (0.02–3.3)	− 0.37	16	1.63 (0.93–2.65)	0.61	15	2.08 (1.16–3.42)^a^	1.19	5	1.14 (0.37–2.66)	0.17
Skin excluding basal and squamous	9	0.58 (0.27–1.11)	− 3.44	66	0.72 (0.55–0.91)^a^	− 2.56	59	0.84 (0.64–1.08)	− 1.71	36	0.82 (0.57–1.13)	− 2.27
Breast	7	0.55 (0.22–1.13)	− 3.06	67	0.96 (0.74–1.22)	− 0.28	48	1.05 (0.77–1.39)	0.34	25	1 (0.64–1.47)	− 0.03
Female genital system	6	1.27 (0.47–2.77)	0.68	28	1.08 (0.72–1.56)	0.21	16	0.94 (0.54–1.53)	− 0.14	10	1.08 (0.52–1.99)	0.22
Male genital system	64	0.79 (0.61–1.01)	− 9.1	414	0.92 (0.83–1.01)	− 3.59	268	0.92 (0.81–1.03)	− 3.71	133	0.87 (0.73–1.03)	− 5.57
Prostate	64	0.8 (0.61–1.02)	− 8.62	409	0.92 (0.83–1.01)	− 3.59	265	0.92 (0.81–1.03)	− 3.65	130	0.86 (0.72–1.03)	− 5.88
Urinary system	60	1.92 (1.47–2.47)^a^	15.34	248	1.35 (1.19–1.53)^a^	6.39	175	1.3 (1.11–1.51)^a^	6.19	102	1.26 (1.03–1.53)^a^	6.12
Urinary bladder	39	2.03 (1.44–2.77)^a^	10.52	172	1.51 (1.29–1.75)^a^	5.72	120	1.41 (1.17–1.69)^a^	5.38	78	1.51 (1.19–1.88)^a^	7.56
Kidney and renal pelvis	19	1.69 (1.02–2.64)^a^	4.14	71	1.1 (0.86–1.38)	0.62	48	1.04 (0.77–1.38)	0.27	22	0.82 (0.52–1.25)	− 1.36
Eye and orbit	0	0 (0–7.93)	− 0.25	11	4.12 (2.06–7.37)^a^	0.82	0	0 (0–1.96)	− 0.29	1	0.92 (0.02–5.15)	− 0.02
Brain and other nervous system	3	0.97 (0.2–2.83)	− 0.05	11	0.63 (0.31–1.12)	− 0.65	8	0.66 (0.28–1.3)	− 0.64	4	0.58 (0.16–1.48)	− 0.84
Endocrine system	27	8.18 (5.39–11.91)^a^	12.63	35	1.88 (1.31–2.61)^a^	1.61	11	0.86 (0.43–1.53)	− 0.28	6	0.86 (0.32–1.87)	− 0.28
Thyroid	26	8.68 (5.67–12.71)^a^	12.26	34	2.01 (1.39–2.8)^a^	1.68	10	0.86 (0.41–1.58)	− 0.26	6	0.95 (0.35–2.08)	− 0.08
All lymphatic and hematopoietic diseases	29	1.1 (0.74–1.58)	1.4	157	1.02 (0.87–1.19)	0.33	120	1.06 (0.88–1.27)	1.12	70	1.03 (0.8–1.3)	0.63
Lymphoma	16	1.24 (0.71–2.02)	1.67	87	1.17 (0.94–1.44)	1.23	51	0.95 (0.71–1.25)	− 0.43	40	1.26 (0.9–1.71)	2.36
Hodgkin–extranodal	0	0 (0–164.76)	− 0.01	0	0 (0–29.62)	− 0.01	0	0 (0–46.02)	− 0.01	0	0 (0–96.07)	− 0.01
Non-Hodgkin lymphoma	14	1.15 (0.63–1.93)	0.98	83	1.18 (0.94–1.46)	1.22	51	1 (0.74–1.31)	− 0.03	40	1.32 (0.94–1.79)	2.75
Myeloma	6	1.21 (0.44–2.64)	0.56	18	0.62 (0.37–0.98)^a^	− 1.08	21	0.97 (0.6–1.48)	− 0.1	15	1.12 (0.63–1.85)	0.47
Lymphocytic leukemia	2	0.45 (0.05–1.64)	− 1.29	18	0.7 (0.41–1.1)	− 0.77	17	0.9 (0.53–1.44)	− 0.28	8	0.73 (0.31–1.43)	− 0.87
Non-lymphocytic leukemia	5	1.21 (0.39–2.82)	0.46	34	1.4 (0.97–1.95)	0.95	31	1.68 (1.14–2.39)^a^	1.93	7	0.6 (0.24–1.24)	− 1.33
Mesothelioma	1	1.06 (0.03–5.89)	0.03	9	1.62 (0.74–3.07)	0.34	0	0 (0–0.91)^a^	− 0.62	5	2.12 (0.69–4.94)	0.76
Miscellaneous	17	1.49 (0.87–2.39)	2.99	115	1.7 (1.4–2.04)^a^	4.66	81	1.59 (1.26–1.98)^a^	4.61	44	1.42 (1.03–1.9)^a^	3.71

^a^p value < 0.05

## Discussion

In our previous work, we characterized the causes of death among patients diagnosed with LC in the USA. One particular observation was the sheer number of deaths attributed to SPMs after LC diagnosis [5]. We hypothesize that this number is rather inflated because of the possible coding and reporting bias of the SEER database itself. For example, the death of an LC patient might be attributed to a site of distant metastasis rather than the index cancer. In fact, this possible error is recognized by the SEER program which lead to the development of a cause-specific survival recode that takes into account various factors including the number of primary tumors per patient, index cancer site, and patients’ comorbidities. Nevertheless, the incidence of developing SPMs in LC is worthy of investigation and has direct impact on survival and quality of life.

In this report, we unraveled the increasing risk of SPMs after LC diagnosis. All patients across different demographic and treatment subgroups demonstrated a statistically significant increased risk of SPM development, except for a few histological subgroups, possibly because of the small number of patients in these groups. Most SPMs were solid tumors anatomically attributed to the head and neck, airway tract, digestive tract, male genital system, and urinary system. Additionally, this work highlights the persistently elevated risk of SPMs in long-term survivors, as well as the temporal trends in SPM development.

The elevated risk of SPMs after LC diagnosis has been previously described in the literature in several studies. However, these studies were often outdated, does not resemble the recent changes in LC trends, management, surveillance, and primary prevention strategies, included only a few patients, or simply did not provide a detailed analysis of the incidence of all possible individual SPM sites. Perhaps the most prominent analysis on this topic was the work of Gao et al., published in 2003, where they used the SEER database to explore the incidence of SPMs in 20,074 patients diagnosed with LC between 1973 and 1996 [8]. Compared to our analysis, the observed numbers of SPMs and SIRs in their study were mostly lower than that reported in our cohort. For example, the incidence of all SPMs in their study was 1.68 compared to our reported value of 2.12, reflecting the possible influence of sample size and the more recent era of diagnosis. Certain anatomical sites, such as the colon and rectum, and the urinary system, failed to reach statistical significance in their study, supporting this assumption. Interestingly, they observed a significant decrease in survival in patients with SPMs and a possible role of radiotherapy as a culprit risk factor [8]. To further emphasize the importance of an updated analysis, Morris et al. reported a significant rising trend in the risk of developing SPMs in LC patients from 1975 to 2006, highlighting the need for a more comprehensive analysis of SPMs risk factors and trends, in light of the poor survival outcomes associated with SPMs diagnosis [9]. Additionally, they studied the SIRs of second solid primaries after the diagnosis of different head and neck index cancers. In their study, the SIRs for oral cavity primaries, oropharynx primaries, larynx primaries, and hypopharynx primaries were 2.82, 2.99, 1.92, and 3.47, respectively. Another study by Ozdemir et al. considered the SPMs as a competing risk of death in LC patients treated with definitive radiotherapy with 2.8% annual incidence. Notably, lung carcinoma emerged as the most frequent type of SPM, with over 50% of these cases were potentially treatable with curative intent, emphasizing the importance of interventions on SPMs for improved long-term outcomes [10].

The causes of SPMs’ development generally fall within three attributable causes: shared genetic susceptibility, shared exposures and environmental risk factors, and possible adverse effects of treatment protocols for the index cancer. For example, the term “field cancerization” was proposed in 1953, referring to tumors with linked anatomical sites due to possible long-term exposure to risk factors, such as tobacco smoking and drinking [11]. Furthermore, the common genetic basis between primary and co-occurring tumors was previously described; most prominently mutations in the P53 gene [12, 13]. The evidence of smoking as a risk factor for the development of SPMs is clinically supported by the rising trends of SPMs in patients diagnosed with smoking-related cancers and their corresponding SPM sites, emphasizing the importance of long-term surveillance in these vulnerable groups [14]. Additionally, several studies have associated the therapeutic exposure to radiotherapy in LC patients with an increased risk of developing SPMs in some patients subgroups [8, 15]. However, we observed an increased risk of SPMs compared to the general population across all treatment stratification in our cohort, including those who refused radiotherapy. Given the limitation in SEER variables, we could not properly assess the risk associated with radiotherapy, as “unknown treatment status” is grouped with “patients who did not receive radiotherapy”, which might introduce inaccuracies. Future studies should assess the impact of SPM development on the overall survival of LC patients and their treatment options. Additionally, given the linkage between the risk factors for different cancer sites, investigating the incidence and outcomes of LC as an SPM in patients diagnosed with different primaries is warranted.

## Conclusions

To conclude, LC Patients are at a significantly increased risk of developing SPMs compared to the general population regardless of their demographics, tumor characteristics, and treatments received. The most common SPMs in this cohort were head and neck, respiratory, digestive, and urinary cancers. Long-term screening and surveillance as well as dedicated prevention programs are advised to enhance the rate of early cancer detection, and control cancer risk factors after LC diagnosis.

## Data Availability

All analyzed data are provided within the manuscript. Public access to the SEER database is available through https://seer.cancer.gov/.

## Abbreviations

SPM: Second primary malignancy
LC: Laryngeal cancer
SEER: Surveillance, epidemiology, and end results
SIR: Standardized incidence ratio
AER: Absolute excess risks
O/E: Observed-to-expected ratio
CI: Confidence interval
